# Fermentation regulation and identification of short-chain and long-chain menaquinones (vitamin K2) from *Elizabethkingia meningoseptica*

**DOI:** 10.3389/fmicb.2026.1865532

**Published:** 2026-07-17

**Authors:** Hongfei Wei, Liping You, Tianjiao Shen, Jiamei Gao, Xiao-dan Liang, Honghan Zhao, Jingru Sun, Zhiping Wu, Xiaohong Li, Chengshi Ding

**Affiliations:** 1 College of Life Sciences, Zaozhuang University, Zaozhuang, China; 2 Department of Clinical Laboratory, Zibo Central Hospital, Zibo, China

**Keywords:** *Elizabethkingia meningoseptica*, fermentation regulation, identification, long-chain menaquinones, short-chain menaquinones

## Abstract

Menaquinone (MKs) are a group of naphthoquinone derivatives that play important roles in various aspects of human health. This study aimed to investigate the congener profiles and yields of MKs produced by *Elizabethkingia meningoseptica* strain Fm, F41, and Fmlv under different cultivation conditions. Notably, the total MK production (three congeners: MK-4, MK-5, and MK-6) by Fm and F41 initially peaked at pH 6. In contrast, Fmlv achieved its maximum MK yield at pH 8 and exclusively produced MK-6. An agitation speed of 140 rpm was found to be optimal for MK production; Fm produced MK-5 and MK-6, whereas F41 produced all three congeners. In contrast, Fmlv showed peak MK production at 160 rpm. Temperature optimization revealed maximal total MK yields for Fm, F41, and Fmlv at 32 °C, with Fmlv demonstrating broader biosynthetic capacity by producing four MK congeners simultaneously. The newly identified MK-7 was further characterized. This is the first study reporting a single *E. meningoseptica* strain capable of synthesizing four different MKs within one fermentation cycle. Altogether, the findings suggest that elevated pH, dissolved oxygen concentration, and temperature during *E. meningoseptica* fermentation selectively enhance the biosynthesis of long-chain MKs, whereas reduced levels of these parameters favor the accumulation of short-chain MK.

## 1 Introduction

Vitamin K2, also referred to as menaquinones (MKs), represent a molecular group with varying side chain length. Different MK forms are denoted as MK-n (*n* = 1–14), where n represents the number of isoprene units in the isoprenoid “tail” (Nowicka and Kruk, 2010). Among them, MK-4 is the most common short-chain form and is produced in human and animal tissues by conversion from vitamin K1. In contrast, the long-chain MKs (MK-5–MK-14) are synthesized exclusively by bacteria (Walther et al., 2013). MKs are essential compounds that humans cannot produce in sufficient quantities and must obtain through diet (Chatterjee et al., 2023; Brandenburg et al., 2015). Reportedly, MKs can outperform vitamin K1 in the context of osteoclastogenesis, hypocholesterolemic effects, and alleviating atherosclerotic progression and bone fracture risk (Fusaro et al., 2022; Brandenburg et al., 2015; Capozzi et al., 2020). They also show the potential for treating mitochondrial disorders such as Parkinson’s disease and amyotrophic lateral sclerosis (Bhalerao and Clandinin, 2012). Notably, some long-chain MK forms, such as MK-7 and MK-9 can present longer plasma half-lives than short-chain MK, such as MK-4, suggesting advantages of their uptake and utilization by the human body (Schurgers and Vermeer, 2002; Sato et al., 2012). Furthermore, dietary MK-7, registered by the US Food and Drug Administration and approved for sale by the European Food Safety Authority, has been associated with reduced coronary heart disease risk (Berenjian et al., 2015; Gast et al., 2009). Significant functional differences exist among different MKs. For instance, MK-4, MK-5, and MK-6 excel in promoting blood coagulation (Huang, et al., 2015), whereas MK-7 and MK-8 can better prevent osteoporosis (Reuters, 2008; Forli et al., 2010). However, these compounds are not readily available and remain costly owing to challenges in their production and purification (Berenjian et al., 2015; Ebrahiminezhad et al., 2016). These challenges necessitate expanding MK production and types to improve the utilization efficiency of different MK homologs according to personalized needs.

Main dietary sources of long-chain MKs originate from bacterial fermentation by species such as *Escherichia coli*, *Lactococcus cremoris*, *L. lactis*, *Bacillus*, and *Flavobacterium* (Dairi, 2023; Liu et al., 2022; Zhang et al., 2025; Berenjian et al., 2015). Among these, A *Flavobacterium* strain can produce one or two among MK-4, MK-5, MK-6, and MK-7, offering potential for multiple MKs (Ren et al., 2020; Kim et al., 2023; Wei et al., 2018a). Hence, this study aimed to investigate MK production and profiles produced by *Elizabethkingia meningoseptica* (formerly classified as *Flavobacterium meningosepticum*) under varying growth conditions (including different growth temperatures, degrees of aeration, and initial pH). Ultimately, an *E. meningoseptica* mutant strain was designed with the aim of simultaneously producing multiple MKs. MKs derived from the mutant were further characterized via mass spectrometry, mid-infrared (IR) spectroscopy, and proton nuclear magnetic resonance (^1^H-NMR) analysis.

## 2 Materials and methods

### 2.1 Bacterial stains and medium

The MK-producing *E. meningoseptica* strains F41 and Fmlv were derived from mutagenesis of the parental strain Fm, subsequently isolated, and maintained at −80 °C. The seed culture medium contained 2% glycerol, 1% fish-derived peptone, 0.15% yeast extract, 0.45% K_2_HPO_4_, 0.3% NaCl, and 0.03% MgSO_4_⋅7H_2_O, with an initial pH adjusted to 7.0 before autoclaving at 121 °C for 20 min. The strains were inoculated at a 2% inoculum in a 500-mL Erlenmeyer flask containing 100 mL of the same medium and aerobically cultured at 37 °C and 120 rpm.

### 2.2 Culture conditions

#### 2.2.1 The effects of initial pH on MK synthesis by different *E. meningoseptica* strains

Strains Fm, F41, and Fmlv were cultivated in media with initial pH gradients of 5, 6, 7, 8, and 9 (other components identical to those of the seed medium), with three replicates per gradient. After 6 d of cultivation at 37 °C and 120 rpm, cells from the fermentation broth were centrifuged at 13,000 *g*, freeze-dried, and extracted with methanol. Finally, MK homolog types and contents were quantified by high-performance liquid chromatography (HPLC).

#### 2.2.2 The effects of dissolved oxygen on MK synthesis by different *E. meningoseptica* strains

To assess dissolved oxygen effects on MK production by different *E. meningoseptica* strains, the dissolved oxygen content was modulated in the medium by controlling the shaking speed of the shaker. Fm, F41, and FmLv were cultured at five speed gradients of 100, 120, 140, 160, and 180 rpm at 37 °C for 6 d, with three replicates per speed gradient. Post-cultivation, fermentation broth was processed as described above, and MK homolog types and contents were quantified by HPLC.

#### 2.2.3 The effects of temperature on MK synthesis by different *E. meningoseptica* strains

Strains Fm, F41, and Fmlv were grown at five temperature gradients of 30 °C, 32 °C, 34 °C, 36 °C, and 38 °C, with three replicates per gradient, in a 120-rpm shaker for 6 d. Fermentation broth handling and MK detection were performed according to the methods described in Section “2.2.1 The effects of initial pH on MK synthesis by different *E. meningoseptica* strains.”

### 2.3 Biomass and MKs quantification

Following cultivation, bacterial cells were processed as previously described (Wei et al., 2018b). Specifically, 25 mL of fermentation broth was centrifuged at 12,000 rpm for 15 min in a 50-mL tube, followed by lyophilization for 24 h. Biomass accumulation was quantified by determining dry cell weight (DCW), as follows:

DCW = (total weight of the centrifuge tube with dry cells) − (weight of the empty centrifuge tube)

### 2.4 Extraction and purification of the MKs from the fermentation medium

Menaquinone were extracted twice from freeze-dried cells using methanol (6:1 v/m ratio, 30 min each), as described previously (Wei et al., 2018b). Their separation and purification were performed on columns employing adsorption chromatography, gel permeation chromatography, and reverse-phase C18 chromatography (Wei et al., 2018a).

### 2.5 Determining the content of MKs and statistical analyses

Methanol extracts of MKs were filtered through a 0.22-μm microporous membrane, and the obtained MK samples were analyzed at 248 nm and 35 °C on the 1260 HPLC system (Agilent, USA) equipped with an ultraviolet (UV)–visible detector and RP C18 column (4.6 mm I.D. × 250 mm). The mobile phase contained methanol:dichloromethane (4:1 v/v) and was used at a flow rate of 1.0 mL/min. MKs were quantified using peak areas and the MK-4 standard curves via the external standard method. All samples from different culture conditions were analyzed in triplicate; data were processed using the SPSS 19 software and reported as mean ± standard deviation. Statistical significance was defined as (*P* < 0.05).

### 2.6 Identification of the MK homologs

Mass spectrometry was performed using an LC-APCI-MS system equipped with an LTQ Orbitrap XL ETD analyzer (Thermo Fisher, Waltham, MA, USA) under the following conditions: APCI^++^ capillary current, 4.0 μA; vaporizer temperature, 300 °C; capillary voltage, 35 V; capillary temperature, 275 °C; sheath gas flow rate, 20.0 arb; and aux gas flow rate, 5.0 arb. Additionally, mid-IR spectra of MKs were recorded on a Bruker Optics Fourier-Transform Infrared spectrometer (ALPHA-T, Bruker Optik GmbH, Germany) at a resolution of 4 cm^–1^ and 64 scans per sample. Lastly, ^1^H-NMR analysis was performed on a Bruker Avance III 600MHz NMR spectrometer (Bruker) at 298 K. Samples were dissolved in deuterated chloroform, with tetramethylsilane as an internal standard.

## 3 Results

### 3.1 Impact of initial pH on MK synthesis

**FIGURE 1:**
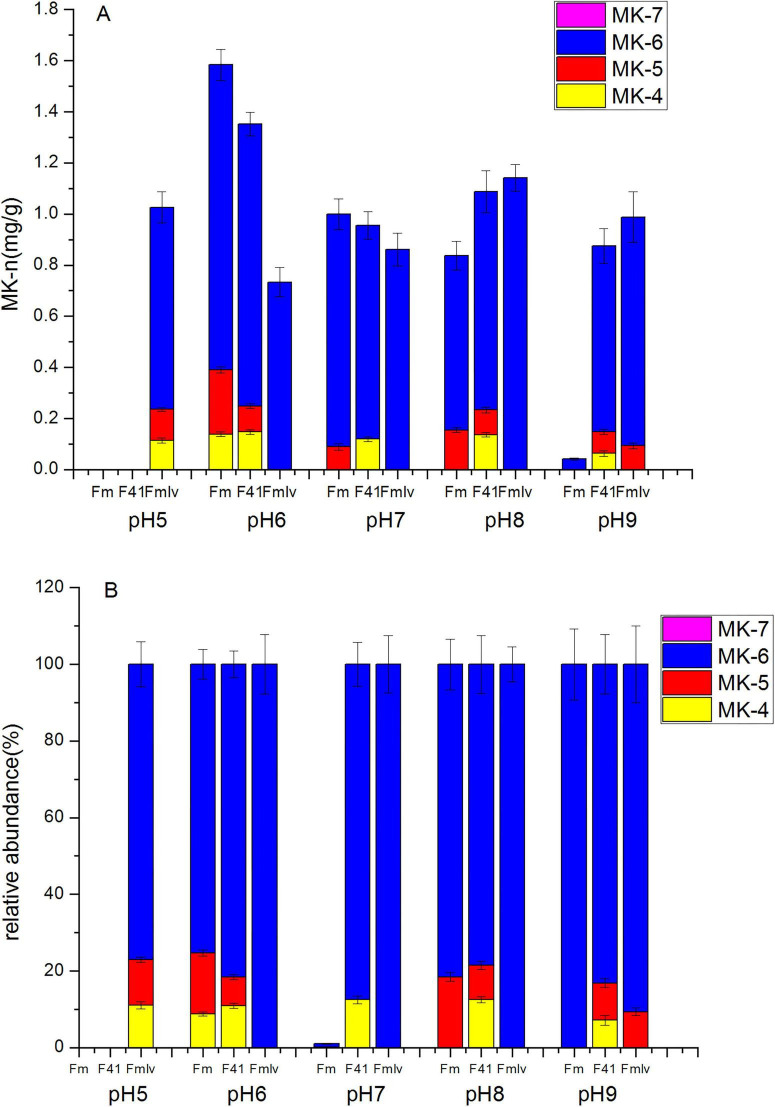
The yields **(A)** and relative abundances **(B)** of MKs produced by *Elizabethkingia meningoseptica* strains Fm, F41, and Fmlv across a range of initial pH values. MK, menaquinone. Three biological replicates were performed for each strain (Fm, F41, and Fmlv) across all initial pH gradients (pH 5–9). Extraction was carried out twice with methanol (6:1 v/m ratio, 30 min each) at room temperature.

Strains Fm, F41, and Fmlv were cultured in fermentation media at initial pH values of 5–9 to assess effects on MK production, followed by analysis of MK types and yields. Notably, strain Fm produced no detectable MKs at pH 5 (Figure 1A), whereas both the yield and diversity of MKs decreased with an increase in pH from 6 to 9. The maximum MK yield for Fm was achieved at pH 6 (1.58 ± 0.08 mg/g, equivalent to 21.44 ± 1.09 mg/L), with MK-4 (8.8%), MK-5 (15.9%), and MK-6 (75.3%) being simultaneously produced (Figure 1B). At pH 7 and 8, only MK-5 (8.9% and 18.5%, respectively) and MK-6 (91.1% and 81.5%, respectively) were produced (Figure 1B). In contrast, culture at pH 9 yielded only MK-6, and the overall yield was markedly reduced. Strain F41 exhibited a similar trend for total MK production compared with that in Fm, with no MK detected at pH 5 and a peak observed at pH 6 (1.35 ± 0.07 mg/g, equivalent to 18.31 ± 0.95 mg/L) (Figure 1A). However, the MK congener distribution was different in F41, with MK-4 and MK-6 being the two MK produced at pH 7, and all three congeners (MK-4, MK-5, and MK-6) being produced under slightly acidic (pH 6) or alkaline (pH 8 and 9) conditions. At the optimal pH 6, MK-4, MK-5, and MK-6 constituted 10.9%, 7.5%, and 81.6%, respectively (Figure 1B). Strain Fmlv showed a broader pH adaptability and produced MKs across pH 5–9. Notably, at near-neutral pH (6–8), Fmlv only produced MK-6, with yields gradually increasing with pH and peaking at pH 8 (1.14 ± 0.05 mg/g, equivalent to 15.46 ± 0.68 mg/L). At pH 5, Fmlv produced MK-4, MK-5, and MK-6 (three types), whereas at pH 9, it only produced MK-5 and MK-6. Despite the higher congener diversity at pH 5, MK yield at pH 5 was lower than that achieved at pH 8.

### 3.2 Influence of dissolved oxygen on MK synthesis

**FIGURE 2:**
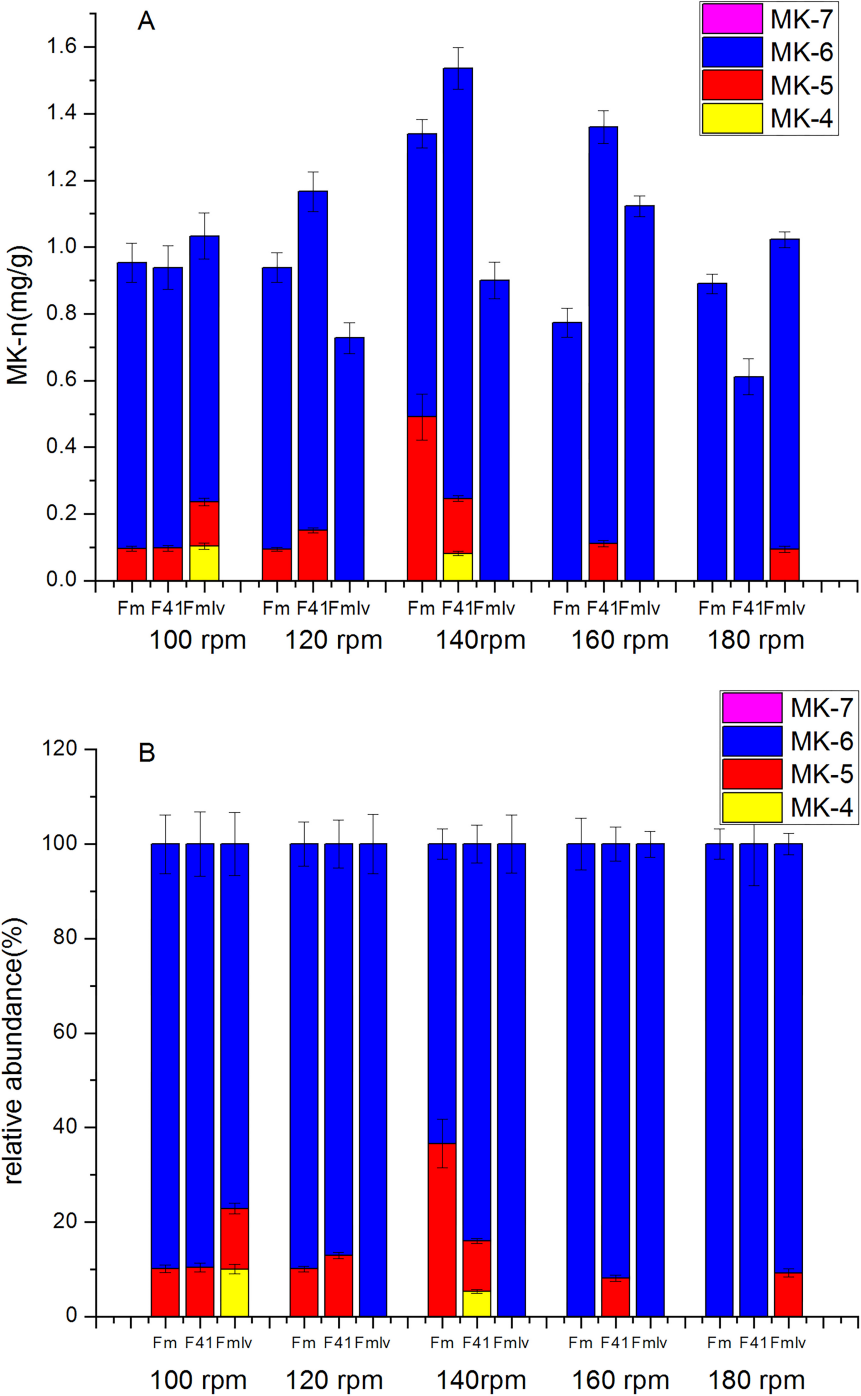
The yields **(A)** and relative abundances **(B)** of MKs produced by *Elizabethkingia meningoseptica* strains Fm, F41, and Fmlv under varying agitation speeds. MK, menaquinone. Three replicates were conducted for each strain (Fm, F41, and Fmlv) across the rotational speed gradient ranging from 100 to 180 rpm. Extraction conditions were the same as in Figure 1.

Dissolved oxygen content in the culture media was controlled by adjusting the agitation speeds (100, 120, 140, 160, and 180 rpm) to investigate its influence on MK production by strains Fm, F41, and Fmlv, with three replicates per condition. Notably, MK production by both Fm and F41 peaked at 140 rpm (1.34 ± 0.11 mg/g and 1.54 ± 0.07 mg/g, equivalent to 18.18 ± 1.49 mg/L and 20.89 ± 0.95 mg/L, respectively) (Figure 2A). Fm produced MK-5 and MK-6 within the range of 100–140 rpm, and only MK-6 was detected at higher speeds (160–180 rpm). In contrast, strain Fmlv exhibited a more complex response to agitation speed. At 100 rpm, Fmlv produced three MKs (MK-4: 10.1%, MK-5: 12.8%, and MK-6: 77.1%), whereas at 180 rpm, only MK-5 (9.3%) and MK-6 (90.7%) were detected (Figure 2B). Notably, at 120–160 rpm, Fmlv exclusively synthesized MK-6, achieving the maximum yield of 1.12 ± 0.03 mg/g, equivalent to 15.19 ± 0.41 mg/L at 160 rpm (Figure 2A). Overall, these results show that the MK profile and yield by *E. meningoseptica* strains can be modulated via precise control of agitation speed, which, in turn, controls dissolved oxygen availability.

### 3.3 Effect of temperature on MK synthesis

**FIGURE 3:**
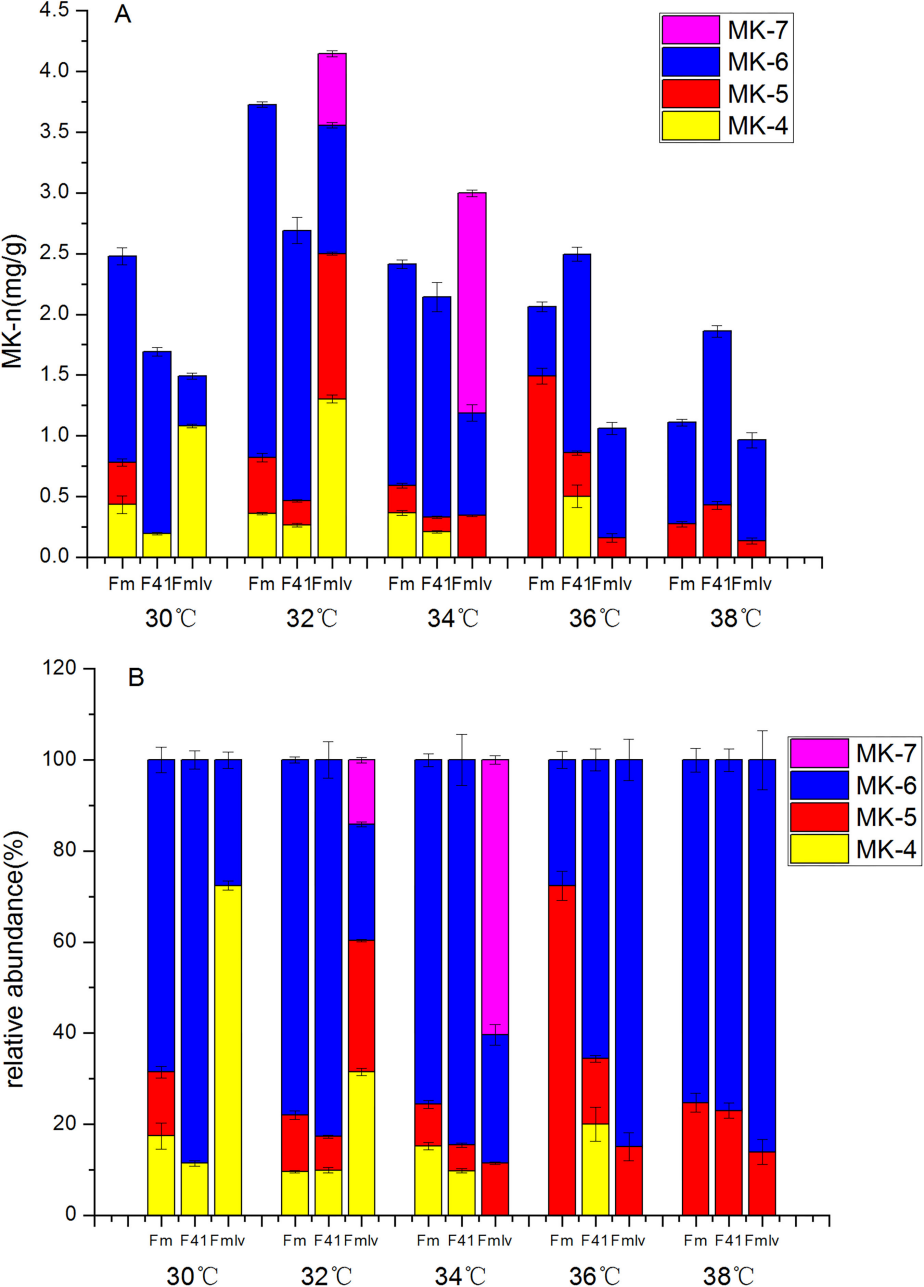
The yields **(A)** and relative abundances **(B)** of MKs produced by *Elizabethkingia meningoseptica* strains Fm, F41, and Fmlv across a range of cultivation temperatures. MK, menaquinone. Three biological replicates were performed for each strain (Fm, F41, and Fmlv) across the temperature gradient ranging from 30 °C to 38 °C. Extraction conditions were the same as in Figure 1.

*Elizabethkingia meningoseptica* strains Fm, F41, and Fmlv were cultivated at 30 °C, 32 °C, 34 °C, 36 °C, and 38 °C (three replicates per condition) to evaluate the effects of temperature. Notably, Strain Fm produced three MK congeners (MK-4, MK-5, and MK-6) at 30 °C–34 °C, which shifted to two congeners (MK-5 and MK-6) at 36 °C–38 °C (Figure 3A). The maximum MK yield from Fm was achieved at 32 °C (3.73 ± 0.07 mg/g, equivalent to 50.60 ± 0.95 mg/L), with MK-4, MK-5, and MK-6 accounting for 9.6%, 12.4%, and 78.0% of total MKs, respectively (Figures 3A, B). In contrast, strain F41 produced MK-4 and MK-6 at 30 °C, whereas at 32 °C–36 °C, three congeners (MK-4, MK-5, and MK-6) were produced. The total MK yield gradually decreased with increasing temperature, with the highest total yield of 2.69 ± 0.13 mg/g, equivalent to 36.50 ± 1.76 mg/L observed at 32 °C, constituting 9.9% MK-4, 7.4% MK-5, and 82.7% MK-6 (Figures 3A, B). Regarding strain Fmlv, total MK production exhibited a biphasic trend across the tested temperature range, where the production initially increased and then decreased. Maximum yield was achieved at 32 °C (4.15 ± 0.09 mg/g, equivalent to 56.30 ± 1.22 mg/L), with four MK homologs, namely MK-4 (31.5%), MK-5 (28.8%), MK-6 (25.5%), and MK-7 (14.2%), being produced simultaneously (Figures 3A, B). In contrast, at 34 °C, Fmlv produced the following three homologs: MK-5 (0.34 ± 0.007 mg/g), MK-6 (0.84 ± 0.068 mg/g), and MK-7 (1.81 ± 0.029 mg/g) (Figure 3A). MK-7 was not detected under any other temperature conditions. To the best of our knowledge, this is the first study to report a mutagenized *E. meningoseptica* strain that can simultaneously produce four MK homologs within a single strain. The total MK yield achieved by this strain Fmlv exceeds that of both the parental wild-type and previously reported mutant strains of *F. meningosepticum*, though it remains marginally lower than the reported MK productivity of *E. meningoseptica* (Liu et al., 2024).

### 3.4 Identification and characterization of MK-7

**FIGURE 4:**
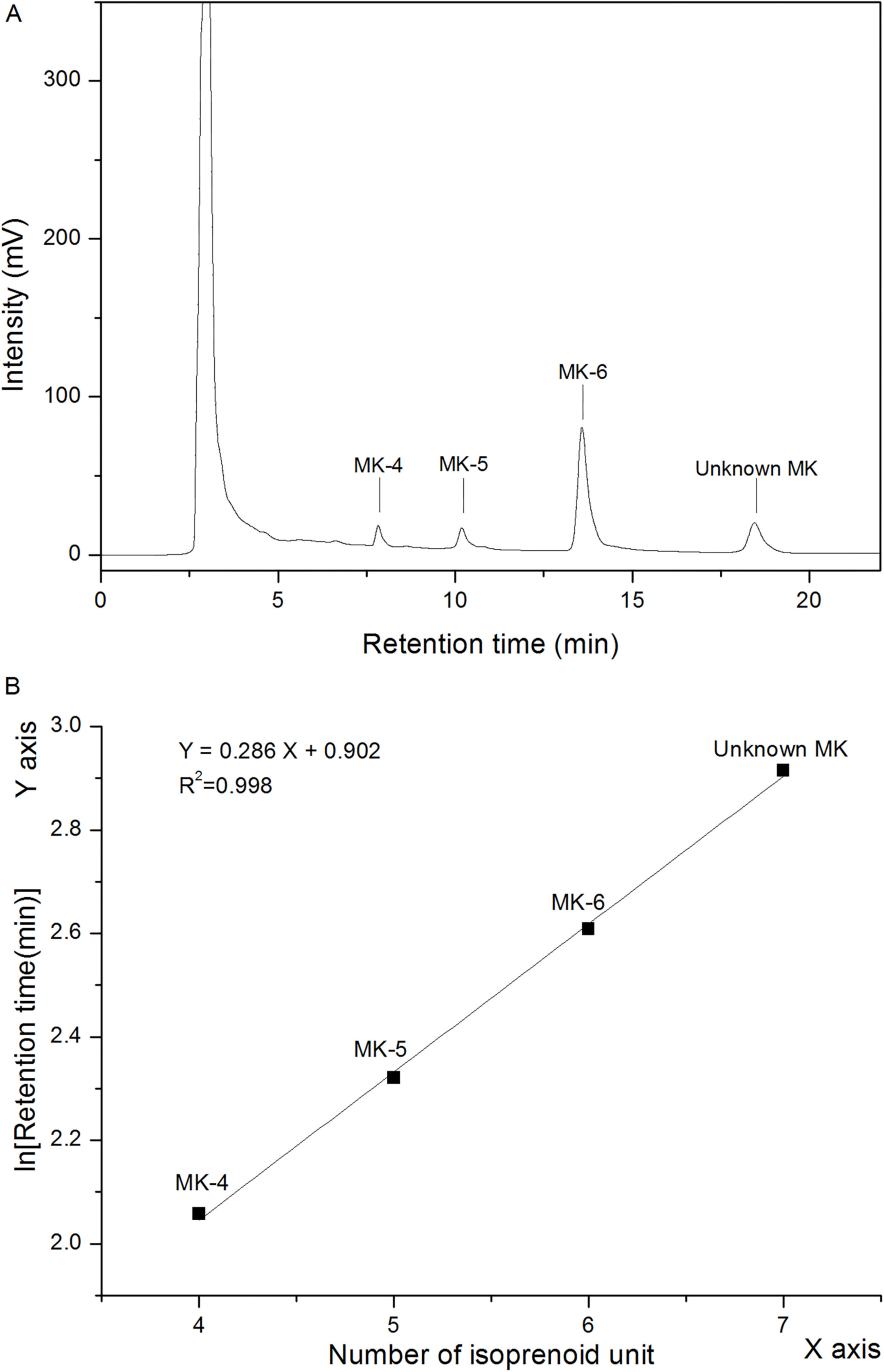
**(A)** HPLC chromatogram of MKs isolated from dry *Elizabethkingia meningoseptica* strain Fmlv. **(B)** ln[retention time] versus isoprene unit number for MK homologues isolated from *Elizabethkingia meningoseptica* strain Fmlv. MK, menaquinone.

Menaquinone species were identified based on HPLC retention time (*Rt*) and their peaks in UV spectra. Additionally, the linear relationship between the logarithm of *Rt* of quinones and isoprenoid units was used to identify quinone species (Irvan et al., 2006). HPLC chromatograms of authentic and isolated MK homologs (Figure 4A) and the straight-line plot between *Rt* on HPLC and isoprenoid units of MK homologs (Figure 4B) showed a quantitative relationship, which is consistent with previously reported findings (Kim et al., 2023). The *Rt* of the newly detected MK homolog corresponded exactly to that of MK-7.

**FIGURE 5:**
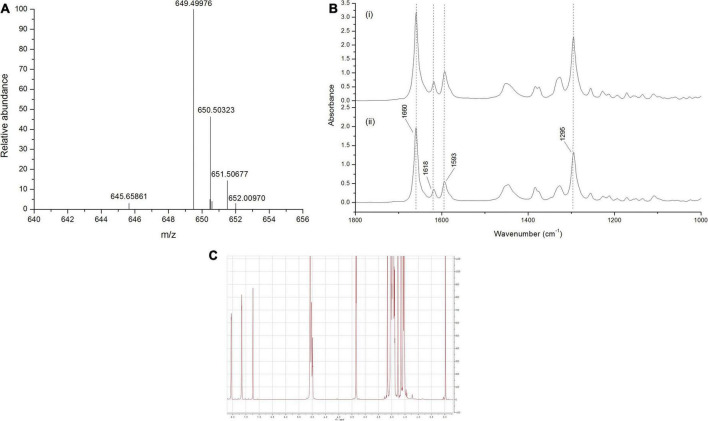
**(A)** Mass spectra of menaquinone (MK)-7 isolated from *Elizabethkingia meningoseptica*. Mass spectrometry conditions: APCI^+^ capillary current, 4.0 μA; vaporizer temperature, 300 °C; capillary voltage, 35 V; capillary temperature, 275 °C; sheath gas flow rate, 20.0 arb; and aux gas flow rate, 5.0 arb. **(B)** Infrared absorption spectra of (i) the MK-4 standard and (ii) MK-7 samples prepared by mixing 2 mg of MK with 200 mg of dried KBr, followed by pressing under 15 MPa pressure for 3 min to make a disk pellet. **(C)** Proton nuclear magnetic resonance spectra of MK-7 purified from *Elizabethkingia meningoseptica*. The MK-7 sample was dissolved in deuterated chloroform with tetramethylsilane as an internal standard and detected at 298 K.

Mass spectra revealed an m/z value of 649 for MK-7, indicating its detection as H^+^-associated ion species and correspondence to MKs (Figure 5A). Mid-IR spectra revealed the split ν (C=O) mode at 1660 cm^–1^, the ν (C=C) mode of the quinoid ring at 1618 cm^–1^, and that of the aromatic ring at 1593 cm^–1^. Notably, the mode at 1295 cm^–1^ denoted a coupled C-C/C=C vibration (Figure 5B). Overall, these characteristic absorption peaks of the tested sample showed consistency with those of MK-4 standard, indicating that the sample was most likely an MK homolog. The ^1^H-NMR data of MK-7 was consistent with the results of mass spectrometry (Figure 5C). Notably, the following absorptions were observed: δ 7.66–8.06 region, denoting four aromatic protons in the quinoid ring (C-7 or C-8 and C-6 or C-9, respectively); chemical shift at δ 5.03, the presence of -CH=C-. Furthermore, the spectra revealed a doublet at δ 3.35 and a singlet at δ 1.77, denoting a methylene and methyl group adjacent to the ring system, respectively, and confirming that the first isoprene unit was unsaturated. A singlet at δ 1.65 was attributed to *cis*-end of chain methyl, indicating that the terminal unit was unsaturated. Lastly, complex absorption trends were observed in the δ 1.88–1.96 and δ 1.52–1.57 regions owing to the presence to isoprene unit and methyl attach to the isoprene unit.

## 4 Discussion

Functional differences among MKs drive their distinct applications and market demands. Notably, long-chain MKs–including MK-5 through MK-14–exhibit significantly extended *in vivo* half-lives relative to short-chain variants (e.g., MK-1 to MK-4), thereby are especially valued in health supplements and pharmaceuticals. *E. meningoseptica* typically produces one or two types of MKs. And, the research on optimization of the profiles of MK congeners or on investigation of the environmental factors influencing their biosynthesis remains limited. In the present study, the production and congener distribution of MKs (MK-4–MK-7) in mutagenized strains of *E. meningoseptica* were assessed under varied conditions. Herein, strain Fmlv showed broader pH tolerance (5–9) compared with that of Fm and F41. Within this range, the yield and congener profile of MKs derived from Fm and Fmlv can be modulated by adjusting the initial pH of the culture medium. Fm was capable of producing short-chain MK-4 and long-chain MKs like MK-5 and MK-6 at pH 6, while predominantly produced long-chain MK-5 and/or MK-6 at pH 7, 8 and 9. Fmlv was capable of producing short-chain MK-4 and long-chain MKs (such as MK-5 and MK-6) at pH 5, while predominantly produced long-chain MK-5 and/or MK-6 at pH 6, 7, 8 and 9. These findings indicated that a lower pH favored short-chain MK-4 synthesis, whereas higher pH conditions promoted the synthesis of long-chain MKs (such as MK-5 and MK-6). A previous study has demonstrated that *Bacillus subtilis* natto exhibits enhanced biomass accumulation and significantly elevated MK-7 biosynthesis under alkaline stress conditions (pH 8.5) (Chen et al., 2022). This implies that alkaline conditions (pH 8.5) promote the biosynthesis of long-chain MK-7, corroborating our experimental observations. Further analysis of MK congener distribution across the three strains revealed a consistent pattern, with MK-6 being the predominant type in all cases.

A previous study has shown that prokaryotes possessing the classical MK biosynthetic pathway–which relies on either the mevalonate (MVA) or methylerythritol phosphate (MEP) pathway for isoprenoid precursor supply–are predominantly aerobic or facultatively anaerobic; in contrast, those utilizing the futalosine pathway encompass aerobic, facultatively anaerobic, and obligately anaerobic lineages (Zhi et al., 2013). *E. meningoseptica* is an aerobic prokaryotic that synthesizes MKs via the MVA pathway. MK is a well-known electron carrier in bacterial respiratory metabolism, particularly when both oxygen and heme are available. Therefore, oxygen effects on MK production and congener distribution under aeration conditions were assessed. At 100–140 rpm, strain Fm produced long-chain MKs like MK-5 and MK-6, but only MK-6 was yielded at 160–180 rpm, indicating that higher oxygen availability favored the biosynthesis of long-chain MKs in this strain. Similarly, strain Fmlv produced short-chain MK-4 and long-chain MKs like MK-5 and MK-6 at 100 rpm; however, MK-4 was not synthesized at 120–180 rpm, indicating the shift toward the production of longer-chain congeners. This pattern is consistent with that observed in strain Fm. However, strain F41 retained the ability to produce MK-4 at 140 rpm, within the range of 100–160 rpm, and increasing agitation speed positively correlated with gradual increase in the total MK-5 and MK-6 contents. At 180 rpm, F41 exclusively synthesized MK-6, suggesting that elevated dissolved oxygen levels promote long-chain MK synthesis in this strain as well. Altogether, these results showed that increasing dissolved oxygen content in the fermentation broth under aerobic metabolic conditions is a viable strategy to enhance the production of long-chain MKs across all three strains. This observation suggests that long-chain MKs are more efficient in the aerobic respiratory transport chain, a result consistent with previous study (Liu et al., 2022).

Temperature significantly affected total yield and congener distribution of long-chain MKs in strains Fm, F41, and Fmlv. Maximum total MK production occurred at 32 °C for all strains. Notably, strain Fm produced MK-4, MK-5, and MK-6 at 30 °C–34 °C, followed by shift to only MK-5 and MK-6 above 34 °C (36 °C or 38 °C), indicating that lower temperatures favor short-chain MK-4 synthesis while higher temperatures promote the synthesis of long-chain MKs (such as MK-5 and MK-6). Although F41 and Fmlv showed similar trends regarding MK-4 synthesis, Fmlv could produce MK-4 up to 36 °C. Notably, neither F41 nor Fmlv produced MK-5 at 30 °C, and it was only synthesized above 32 °C, which further supported that elevated temperatures facilitate long-chain MK biosynthesis. These results are consistent with the findings of Liu et al. (2019), who reported that temperature may modulate the activity of key enzymes involved in MK biosynthesis, specifically those involved in isoprenoid side chain elongation. Notably, Fmlv demonstrated an unprecedented metabolic capability, as it simultaneously synthesized four MK congeners, namely MK-4, MK-5, MK-6, and MK-7, at 32 °C, and three (MK-5, MK-6, and MK-7) at 34 °C. This is the first study reporting the production of four MK congeners by a single *E. meningoseptica* strain during one fermentation process. This finding enables the enhancement of total MK production by expanding the MKs spectrum during fermentation, and also provides a strain resource for metabolic engineering in future studies (Zhang et al., 2021).

## 5 Conclusion

The functional differences among various MKs present notable challenges in the targeted biosynthesis of specific MK types in *E. meningoseptica* through metabolic regulation, which is a significant objective for industrial and biomedical applications. The findings of this study show the congener profiles and yields of MKs produced by different *E. meningoseptica* strains under varied cultivation conditions. The total MK production (comprising MK-4, MK-5, and MK-6) by strains Fm and F41 peaked at an initial pH of 6, whereas strain Fmlv achieved maximum MK yield (MK-6) at pH 8. Under varying agitation speeds, the highest total MK yields were observed at 140 rpm for Fm (MK-5 and MK-6) and F41 (MK-4, MK-5, and MK-6); Fmlv exhibited peak MK production (MK-6) at 160 rpm. Finally, temperature optimization revealed 32 °C yields the maximal total MK content for all three strains, with Fm and F41 producing MK-4, MK-5, and MK-6, and Fmlv demonstrating a broader biosynthetic capacity by simultaneously producing four MK congeners (MK-4, MK-5, MK-6, and MK-7). To the best of our knowledge, this is the first report of a single *E. meningoseptica* strain capable of simultaneously synthesizing four distinct MK forms within one fermentation cycle. These findings indicate that elevated pH, dissolved oxygen concentration, and temperature during *E. meningoseptica* fermentation selectively enhance the biosynthesis of long-chain MKs (such as MK-5, MK-6, and/or MK-7), whereas reduced levels of these parameters favor the accumulation of short-chain MK-4. Altogether, this study contributes to the enhancement of total MK production by expanding the MKs spectrum during fermentation, and also provides a strain resource for metabolic engineering in future studies.

## Data Availability

The original contributions presented in this study are included in the article/supplementary material, further inquiries can be directed to the corresponding authors.
